# Long-term functional swallowing and speech outcomes after transoral robotic surgery for oropharyngeal cancer

**DOI:** 10.3389/fsurg.2024.1362654

**Published:** 2024-01-31

**Authors:** Yong Bae Ji, Hae Won Choi, Chang Myeon Song, Bo Ram Yun, Hae Jin Park, Sukjoong Oh, Kyung Tae

**Affiliations:** ^1^ Department of Otolaryngology-Head and Neck Surgery, College of Medicine, Hanyang University, Seoul, Republic of Korea; ^2^ Department of Radiation Oncology, College of Medicine, Hanyang University, Seoul, Republic of Korea; ^3^ Department of Internal Medicine, College of Medicine, Hanyang University, Seoul, Republic of Korea

**Keywords:** oropharyngeal cancer, transoral robotic surgery, functional outcomes, speech, swallowing, modified barium swallowing

## Abstract

**Objectives:**

Transoral robotic surgery (TORS) has emerged as a minimally invasive approach for oropharyngeal cancer, aiming to improve functional preservation and reduce morbidity. However, the long-term effects on speech and swallowing, crucial aspects of quality of life, remain unclear. This study investigates the long-term functional swallowing and speech outcomes of TORS for oropharyngeal cancer.

**Methods:**

We retrospectively reviewed 41 patients diagnosed with oropharyngeal squamous cell carcinoma who underwent TORS from 2010 to 2018. Tongue mobility, articulation, verbal diadochokinesis, reading speed, and modified barium swallowing tests were performed 2–3 years post-operatively to assess long-term speech and swallowing function.

**Results:**

The mean age was 57.7 ± 9.9 years, and the male to female ratio was 34:7. The palatine tonsil was the most common tumor site (73.2%), followed by the base of tongue (22.0%). Concurrent neck dissection was performed in 97.6% of patients, and adjuvant radiation or chemoradiation was administered to 36 patients (87.8%). Tongue mobility, articulation, verbal diadochokinesis, and reading speed were comparable to normal population. Modified barium swallowing tests revealed acceptable outcomes in most patients; only one patient (2.4%) required a percutaneous endoscopic gastrostomy tube. Notably, no permanent tracheostomies were necessary.

**Conclusions:**

Long-term speech and swallowing functions were preserved in most patients treated with TORS for oropharyngeal cancer. TORS is an excellent treatment modality for oropharyngeal cancer in terms of functional outcomes.

## Introduction

The incidence of oropharyngeal cancer, especially human papillomavirus (HPV)-related oropharyngeal squamous cell carcinoma (OPSCC), is increasing worldwide (1). HPV-positive OPSCC has better treatment outcomes and prognoses compared to HPV-negative cancer because HPV-positive OPSCC generally occurs in younger patients who are more likely to survive longer. Therefore, preservation of function and reduction of morbidity are more critical in treating HPV-positive patients.

The primary treatment for oropharyngeal cancer has been radical excision of the primary tumor using various approaches and subsequent adjuvant radiation treatment. This treatment strategy results in inevitable functional disturbance, especially in speech and swallowing (2). Therefore, based on some landmark studies, organ preservation chemoradiation therapy has been developed and confirmed to be comparable to surgical treatment in avoiding surgical morbidity and functional loss (3, 4). Since then, chemoradiation therapy has been considered the primary treatment for oropharyngeal cancer.

However, long-term results of chemoradiation therapy in treating head and neck cancer were reported, and high dose chemoradiation therapy resulted in significant short- and long-term morbidities such as xerostomia and dysphagia (5–7). A systematic review showed that 10%–30% of patients require gastrostomy one year after treatment. This results in a significantly adverse impact on quality of life (8). Therefore, functional preservation, not merely organ preservation, are important; and treatment outcome and quality of life and function need to be considered when selecting a treatment strategy.

Transoral robotic surgery (TORS) for oropharyngeal cancer was introduced with the aim of better functional preservation with less morbidity, and the United States Food and Drug Administration approved TORS in 2009.

Oncologic outcomes of TORS for oropharyngeal cancer are comparable to those of primary chemoradiation or radical surgery (9). In terms of functional outcome, TORS showed excellent short-term results, including more rapid recovery of swallowing, shorter hospitalization, and shorter operation time (10–12). However, the actual long-term functional outcomes of TORS, including speech and swallowing outcomes, have not been thoroughly assessed, but some studies did evaluate long-term functional outcomes of tracheostomy, feeding tube dependency, and questionnaire-based subjective results after TORS (13–18). Therefore, this study aimed to evaluate long-term functional speech and swallowing outcomes after TORS for oropharyngeal cancer.

## Materials and methods

We retrospectively reviewed data from 63 patients with OPSCC who underwent primary TORS with or without adjuvant radiation or chemoradiation therapy between January 2010 and December 2018. The indication of TORS is small and moderate-sized oropharyngeal cancer without fixation to the lateral pharyngeal wall, prevertebral fascia and carotid artery in our institution. We thoroughly explained surgical and non-surgical treatment options to patients and respected their opinions in making treatment decisions at multidisciplinary team meetings. Of 63 patients, we excluded 22 patients from the study, including those who did not perform a swallowing and speech test between two and three years after TORS (16 patients) and who had a previous history of head and neck surgery or irradiation (1 patient), or who had cancer recurrence within two to three years after TORS before functional evaluation (5 patients). Finally, the remaining 41 patients were included, and data from these patients were analyzed in this study. The study protocol was approved by the institutional review board.

All operations were performed by a single surgeon. TORS was performed using the da Vinci Si surgical system (Intuitive Surgical, Inc., Sunnyvale, CA). FK retractor (Gyrus Medical Inc., Tuttlingen, Germany) or the Crowe-Davis mouth gag were used to expose the oropharynx. The compartment-oriented *en bloc* dissection was performed for the primary tumor of the tonsil or BOT. Simultaneous selective or modified radical neck dissection with TORS for the primary tumor was performed according to the status of lymph node metastasis. Tracheostomy was performed only in patients with suspiciously compromised airways.

We performed adjuvant radiation therapy after TORS in patients with close surgical margin or lymph node metastasis and adjuvant concurrent chemoradiation therapy in patients with positive margin or extranodal extension.

Evaluation of postoperative functional speech and swallowing outcomes was performed by the mobility of the tongue, articulation, verbal diadochokinesis, reading speed, and modified barium swallowing tests conducted between two and three years after surgery. All tests were performed by single experienced speech-language pathologist. Tracheostomy tube or percutaneous endoscopic gastrostomy (PEG) tube dependency was also investigated.

### Assessment of articulation and speech

Speech and articulation functions were evaluated using the Korean Speech Mechanism Screening Test designed to assess the structure and function of articulation and speech compared to the data of the normal Korean population (19).

Tongue mobility was assessed by the limitation of lingual motions (Table 1). Each tongue motion was assessed with a score of 0 (severe impairment), 1 (mild impairment), or 2 (normal). The tongue mobility score was the sum of the 8 lingual motion scores.

**Table 1:** Assessment of tongue mobility by the Korean speech mechanism screening test.

1. Put your tongue out as far as you can. (protrusion)
2. Move your tongue to the left and right corner of your mouth. (protrusion and lateralization)
3. Open your mouth as far as you can and touch your tongue tip to your incisor. (length of frenulum)
4. Move your tongue up to the hard palate. (elevation)
5. Pull your tongue back in your mouth as far as possible. (retraction)
6. Push your lower incisor with your tongue. (elevation and protrusion)
7. Pull your tongue tip as far back in your mouth as you can. (retroflexion)
8. Roll your tongue. (rolling)
Scoring: Normal (0), Mild impairment (1), Severe impairment (2). Range of total score: 0–16

Articulation was evaluated by having the patient read Korean poetry comprising 39 syllables. The speech-language pathologist assessed the accuracy of /r/, /s/, and /z/ pronunciation, assigning scores of 0 (severe impairment), 1 (mild impairment), or 2 (normal). The articulation score was defined as the sum of the 3 consonant scores.

The verbal diadochokinesis test was utilized to assess oral motor function (20). he patients were instructed to pronounce the four sounds /pʌ/, /tʌ/, /kʌ/, /pʌtʌkʌ/, /rʌ/, /gn/, and /a/ as rapidly as possible for a duration of 5 s. This exercise was repeated three times. The speech pathologist assessed the regularity (scored on a scale of 0–14) and accuracy (scored on a scale of 0–14) of each pronunciation, assigning a score of 0 (indicating severe impairment), 1 (mild impairment), or 2 (normal). The verbal diadochokinesis score was defined as the sum of regularity and accuracy score.

Reading speed was evaluated by having the patients read Korean poetry consisting of 60 syllables, and 18.2 s or more was considered abnormal in adults.

### Assessment of swallowing

Swallowing assessment was conducted with the modified barium swallow (MBS) study (21). During the MBS, the patient sits upright on a chair or stands on a platform. Thin liquids were administered to subjects with increasing volume (3, 5, and 10 ml), and food items of different consistencies that have been mixed with barium-sulfate–containing products (Varibar®, Bracco Diagnostics Inc., Monroe Twp., NJ) were also administered. The standard lateral radiographic views from the lips to the cervical spine and from the nasopharynx to the upper esophageal sphincter were obtained. We evaluated swallowing performance in each process of the oral (e.g., tongue mobility and mouth residues), pharyngeal (e.g., triggering of pharyngeal swallow, laryngeal elevation and epiglottic closure and nasal regurgitation) and esophageal (e.g., obstruction, passage, and reflux) phases.

The Dynamic Imaging Grade of Swallowing Toxicity (DIGEST) was used to analyze the MBS (22). It is a validated staging tool to estimate the severity of pharyngeal dysphagia based on the MBS study. The scale comprises two component scores: (1) safety rating and (2) efficiency rating. For the safety scoring, Penetration-Aspiration Scale (PAS) scale score was measured during the MBS (23). For the efficiency rating, the evaluator assessed a maximum percentage of pharyngeal residue in four grades (<10%, 10%–49%, 50%–90%, and >90%). The DIGEST scales an ordinal summary of 5 points based on a value by correlating the parameters of safety grade and efficiency grade: grade 0 (no dysphagia), 1 (mild dysphagia), 2 (moderate dysphagia), 3 (severe dysphagia), 4 (life-threatening).

### Statistical analysis

All analysis was performed using SPSS software (IBM SPSS Statistics for Windows, Version 24.0. Armonk, NY: IBM Corp, USA). Recurrence-free survival was assessed with the Kaplan–Meyer method. Statistical significance was set at *p* values < 0.05.

## Results

The mean age of the 41 patients was 57.7 ± 9.9 years and the male-to-female ratio was 34:7. The most common subsite was the palatine tonsil (30 cases), followed by the base of tongue (9 cases). Pathologic testing confirmed that 38 (92.7%) were squamous cell carcinomas and 3 were basaloid squamous cell carcinoma. Nineteen out of 26 patients (73.1%) were p16 positive. According to the 8th version of the American-Joint Cancer Classification (AJCC) staging system (24), the number of T1/T2/T3/T4 patients was 17/23/1/0, the number of N0/N1/N2 patients was 12/19/10 and the number of stage I/II/III/IV patients was 7/3/19/12. Forty patients (97.6%) underwent neck dissection concomitant with TORS. Tracheostomy was performed on six (14.6%) TORS patients. The mean time of console work for TORS was 94.7 ± 41.5 min. There were 1 case of minor hematoma and 6 cases of seroma in the neck. None of the cases was converted to conventional surgery. Thirty-six patients (87.8%) received adjuvant radiation or chemoradiation therapy (Table 2).

**Table 2:** Clinicopathologic characteristic of patients with oropharyngeal cancer who underwent TORS.

	*N* = 41
Sex (M: F)	34:7
Age (years)	57.7 ± 9.9
Comorbid disease	
Diabetes/Hypertension	8 (19.5%)/18 (43.9%)
Smoking history	
None/Former/Current	17/8/16
Alcohol history	
None/Social/Heavy^a^	15/19/7
Socioeconomic status	
High/Middle/Low	8/27/6
Primary site	
Tonsil	30 (73.2%)
BOT	9 (22.0%)
Soft palate	1 (2.4%)
Pharyngeal wall	1 (2.4%)
Tumor size (mm)	24.6 ± 9.6
Pathology	
SCC	38 (92.7%)
Basaloid SCC	3 (7.3%)
P16+	19/26 (73.1%)
T classification	
T1/T2/T3/T4	17/23/1/0
N classification	
N0/N1/N2	12/14/15
Stage	
I/II/III/IV	7/3/15/16
Adjuvant therapy	35 (85.4%)
Radiation only	18 (43.9%)
Chemoradiation	17 (41.5%)

TORS, transoral robotic surgery; BOT, base of tongue: SCC squamous cell carcinoma.

^a^ Heavy drinker: consuming 3 or more drinks per week.

We defined the abnormal cut-off values for tongue motility, maximal phonation time, verbal diadochokinesis, articulation tests, and reading speed as two standard deviations above or below the value of normal subjects (Table 3).

**Table 3:** Articulation and speech outcomes.

	Mean ± SD	Range	Abnormal reference value^19^ (2 SD of normal subject)
Tongue mobility (0–16)	15.5 ± 1.2	13–17	<14.58
Articulation score (0–6)	5.9 ± 0.3	5–6	<4.79
Verbal diadochokinesis (0–28)	25.1 ± 2.1	22–28	<21.83
Reading speed (s)	11.9 ± 2.3	9.1–15.8	>18.2 s

SD, standard deviation.

The mean tongue motility score was 15.5 ± 1.2 (range, 13–17) in this study. Only 3 patients had abnormal tongue motility scores below the cut-off value defined in the study (below 14.58).

The mean articulation score was 5.9 ± 0.3 (range, 5–6) in this study. No patient showed an abnormal cut-off value score (lower than 4.79). The mean verbal diadochokinesis score (sum of regularity and accuracy) was 25.1 ± 2.1 in this study, which was higher than the abnormal reference value (<21.83). The mean reading speed was 11.9 ± 2.3 s (range, 9.1–15.8) in this study. All patients were below the abnormal cut-off value score (higher than 18.2 s) (19).

In the subgroup analysis of articulation and speech outcomes according to primary site and T classification, there were no significant differences between the tonsil and base of tongue and between T1 and T2 primary cancers (Table 4).

**Table 4:** Comparison of articulation and speech outcomes according to primary sub-site and T classification.

	Tonsil	BOT	*P*	T1	T2	*P*
Tongue mobility (0–16)	15.6 ± 1.2	15.6 ± 1.2	0.681	15.7 ± 0.9	15.6 ± 1.5	0.909
Articulation score (0–6)	5.9 ± 0.2	6.0 ± 0.3	0.600	6.0 ± 0.2	5.9 ± 0.3	0.357
Verbal diadochokinesis (0–28)	25.4 ± 2.4	27.0 ± 2.2	0.212	26.6 ± 1.5	25.5 ± 2.7	0.312
Reading speed (s)	12.1 ± 1.9	10.6 ± 2.6	0.196	12.4 ± 2.2	11.7 ± 2.0	0.520

TORS, transoral robotic surgery; BOT, base of tongue.

MBS was performed in 32 patients. Generally, the results of MBS were favorable in most patients. The most common DIGEST scale was DIGEST 1 (*n* = 22, 68.8%), followed by DIGEST 0 (*n* = 8, 25.0%) and 2 (*n* = 2, 6.3%) (Supplementary Video S1). None of the patients scored DIGEST 3 or 4. In the safety grade, 22 patients (68.8%) showed grade 0 and 10 patients (31.3%) showed grade 1. In the efficiency grade, 20 patients (62.5%) showed grade 1.

In a detailed investigation of swallowing performance, mild piecemeal deglutition was noted in 3 patients, premature bolus loss in 8 patients, and presence of mouth residue in 3 patients in the oral stage. Reduced laryngeal elevation and epiglottic closure and repeated swallowing were seen in only 2 patients in the pharyngeal stage. In the esophageal stage, delayed passage was noted in 7 patients, opening of pharyngoesophageal segment in 4 patients, and gastroesophageal reflux in 4 patients (Table 5). None of the patients needed nasogastric or PEG tubes before adjuvant therapy. However, one patient (2.4%) with T2 tonsil cancer required permanent PEG tube placement after chemoradiation therapy. No patients required permanent tracheostomy.

**Table 5:** Functional outcomes of swallowing as assessed by modified barium swallowing.

	*N* = 32
DIGEST score	
0	8 (25.0%)
1	22 (68.8%)
2	2 (6.3%)
Oral phase	
Lip closure	0
Chewing & mastication	0
Tongue elevation & palate contact	0
Tongue thrust	0
Piecemeal deglutition	3 mild (9.4%)
Premature bolus loss	8 mild (25.0%)
Residue in mouth	3 mild (9.4%)
Pharyngeal phase	
Delayed triggering of pharyngeal swallow	0
Reduced laryngeal elevation & epiglottic closure	2 (6.3%)
Repeated swallow	2 (6.3%)
Nasal regurgitation	0
Coating of pharyngeal wall after swallow	0
Esophageal phase	
Mechanical obstruction	0
Delayed passage	7 (21.9%)
Opening of pharyngoesophageal segment	4 (12.5%)
Gastroesophageal reflux	4 (12.5%)

DIGEST, Dynamic Imaging Grade of Swallowing Toxicity.

## Discussion

Oncologic outcomes after TORS in oropharynx cancer are generally favorable. Also, despite initial concerns about the imperfection of *en bloc* resection, TORS is recognized by many researchers as a relatively safe technique. In a systematic review encompassing 12 TORS studies involving 772 patients, the reported adverse events of TORS included hemorrhage (2.4%), fistula (2.5%), and the placement of gastrostomy tubes at the time of surgery (1.4%). However, the necessity for gastrostomy tubes increased to 30% of patients during adjuvant treatment (25). Another study, which analyzed data from 305 patients in the American College of Surgeons National Surgical Quality Improvement Program (ACS NSQIP) datasets, demonstrated a low complication rate of 7.9% and a 1-month mortality rate of 0.7% (26).

There were no severe complications or mortality in this study, but there were some minor complications such as minor hematoma and seroma. Also, no procedure was interrupted or converted to conventional radical surgery because the tumor could not be removed during TORS.

Several studies reported that the temporary tracheostomy rate was from 0 to 31% (less than 10% in most studies) and the permanent tracheostomy rate was 0%–2% (9, 15, 16, 18). In this study, temporary tracheostomy was performed on six patients during TORS. However, none of these patients required permanent tracheostomy.

Functional outcomes are essential, particularly in HPV-related oropharyngeal cancer, because this cancer occurs in relatively younger patients who respond well to both surgical and non-surgical treatment modalities and show good prognoses. Therefore, post-treatment morbidity, such as xerostomia and dysphagia, can be a life-long problem in these patients. From a functional outcome point of view, primary TORS can be an excellent alternative to concurrent chemoradiation therapy (7).

We assessed speech ability because impaired tongue mobility or velopharyngeal insufficiency can occur when the tongue base or soft palate is resected. To evaluate speech-related function, we used the Korean Speech Mechanism Screening Test, a functional scale specially designed for use in Korea. The test has been validated in the normal Korean population and includes tests for tongue mobility, verbal diadochokinesis, articulation, and reading speed. In this study, long-term functional speech outcomes were acceptable and comparable to those of normal subjects. All speech parameters, including tongue mobility, verbal diadochokinesis, articulation, and reading speed, did not differ from those of the normal population.

Some previous papers also reported favorable speech function after TORS for oropharyngeal cancer as measured by other methods. For example, a study conducted by Moore et al. revealed that all 45 patients who underwent TORS for oropharyngeal cancer showed normal speech function at four weeks postoperatively. In that study, speech was assessed as normal, having minor dysphonia, or having gross dysphonia. However, 4 of that study's patients had rhinolalia when discharged from the hospital (15). Dziegielewski et al. also reported speech function was not different from the preoperative baseline in 76 patients 12 months postoperatively when assessed using a health-related quality of life questionnaire (11).

To analyze swallowing outcomes after TORS, various methods, such as feeding tube rate, fiberoptic endoscopic evaluation, MBS, and swallowing-related quality of life, were used in previous studies. Swallowing function usually declines in immediate postoperative periods and is restored within several weeks (10, 14, 15, 23). In addition, postoperative swallowing outcome is related with preoperative function, T-classification, nodal status, location of primary tumors, and need for adjuvant chemoradiation (15).

A recent questionnaire-based randomized controlled study reported that the swallowing quality of life in the radiation treatment group is higher than in the TORS group (27). However, the perioperative feeding tube rate in TORS is relatively lower than that when using non-surgical therapy (29% to 60%), although it varies from 3% to 100% (10, 14, 15). Sinclair et al. reported that ten out of 42 primary TORS patients with cancer of the oropharynx required gastrostomy tubes. However, this rate improved over time, even after 12 months, and no one required a PEG tube by the commencement of radiation therapy (18). Chronic PEG tube dependence was reported to be from 0 to 7% (14). Sharma et al. reported that stage-matched patients undergoing TORS for oropharyngeal cancer had lower PEG tube dependency compared to patients undergoing non-surgical therapy (33.3% vs. 84.1%), although the PEG tube prevalence decreased over time in both TORS and non-surgical groups (17). In this study, only 1 patient (2.4%) was dependent on a PEG tube at 36 months of follow-up.

In this study, we objectively evaluated swallowing outcomes using MBS. The MBS test was performed in 32 out of 41 patients. Most patients showed favorable swallowing outcome in this study, although there were minor impairments in some patients. No prior research has evaluated swallowing outcome after TORS using MBS. Most previous studies were based on questionnaires, including the University of Washington Quality of Life Questionnaire (12), the EAT-10 (13), and the MD Anderson Dysphagia Inventory (18). In a study comparing 92 patients with early-stage oropharyngeal cancer treated with TORS with/without adjuvant therapy and 46 patients treated with definitive chemoradiation therapy, the two groups showed similar locoregional control rate, overall survival, and disease-free survival. However, the TORS group had a significantly better saliva-related quality of life than the definitive chemoradiation therapy group until 24 months after treatment (12). Achim et al. also reported an adverse effect of adjuvant therapy on swallowing. This group showed that the TORS-only group showed faster restoration of swallowing and less weight loss in the long-term than the TORS with radiation or chemoradiation therapy group (13). However, generally, swallowing function and health-related quality of life deteriorate in the immediate postoperative stage and then gradually recover after TORS regardless of the need for adjuvant therapy (13, 18).

This study has some limitations. First, the design was retrospective in nature, and the sample size was relatively small. Therefore, bias may have been introduced. However, we have routinely collected data on postoperative functional outcomes in head and neck cancer for more than 15 years. Therefore, the reliability and consistency of our data may be adequate. Second, in this study, most patients (85.4%) received adjuvant radiation or chemoradiation therapy after TORS. Therefore, it is a limitation to evaluate functional outcomes after TORS only, excluding the effect of adjuvant treatment. Third, we did not compare the functional results of TORS with those of conventional radical surgery or concurrent chemoradiation therapy. Further comparative studies with larger sample sizes and long-term follow-up are necessary to clearly determine postoperative long-term functional speech and swallowing outcomes after TORS.

## Conclusion

TORS showed favorable long-term functional speech and swallowing outcomes. It can be an excellent treatment modality for oropharyngeal cancer in terms of functional outcomes. Future studies may be mandatory to evaluate the functional outcomes of TORS without adjuvant treatment and compare functional results with non-surgical treatments.

## Data Availability

The raw data supporting the conclusions of this article will be made available by the authors, without undue reservation.
